# Supplementary figures and images for: Katanin, kinesin-13 and ataxin-2 inhibit premature interaction between maternal and paternal genomes in C. elegans zygotes
Source: bioRxiv. 2024 Jun 26:2024.03.12.584242. Originally published 2024 Mar 13. Preprint. [Version 2] doi: 10.1101/2024.03.12.584242 (PMC10979973; doi:10.1101/2024.03.12.584242)

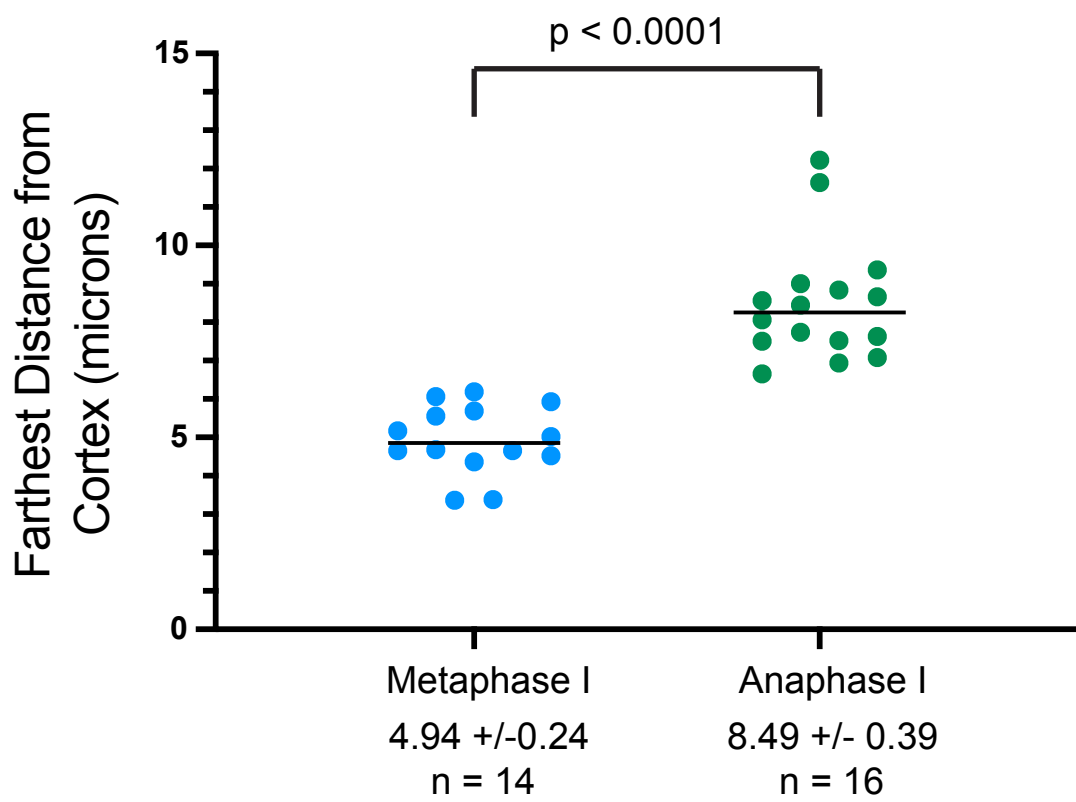

Supplement: Supplement 6 — Figure S1. Distance of the sperm contents from the cortex of control embryos at metaphase I vs anaphase I. [file media-6.pdf]

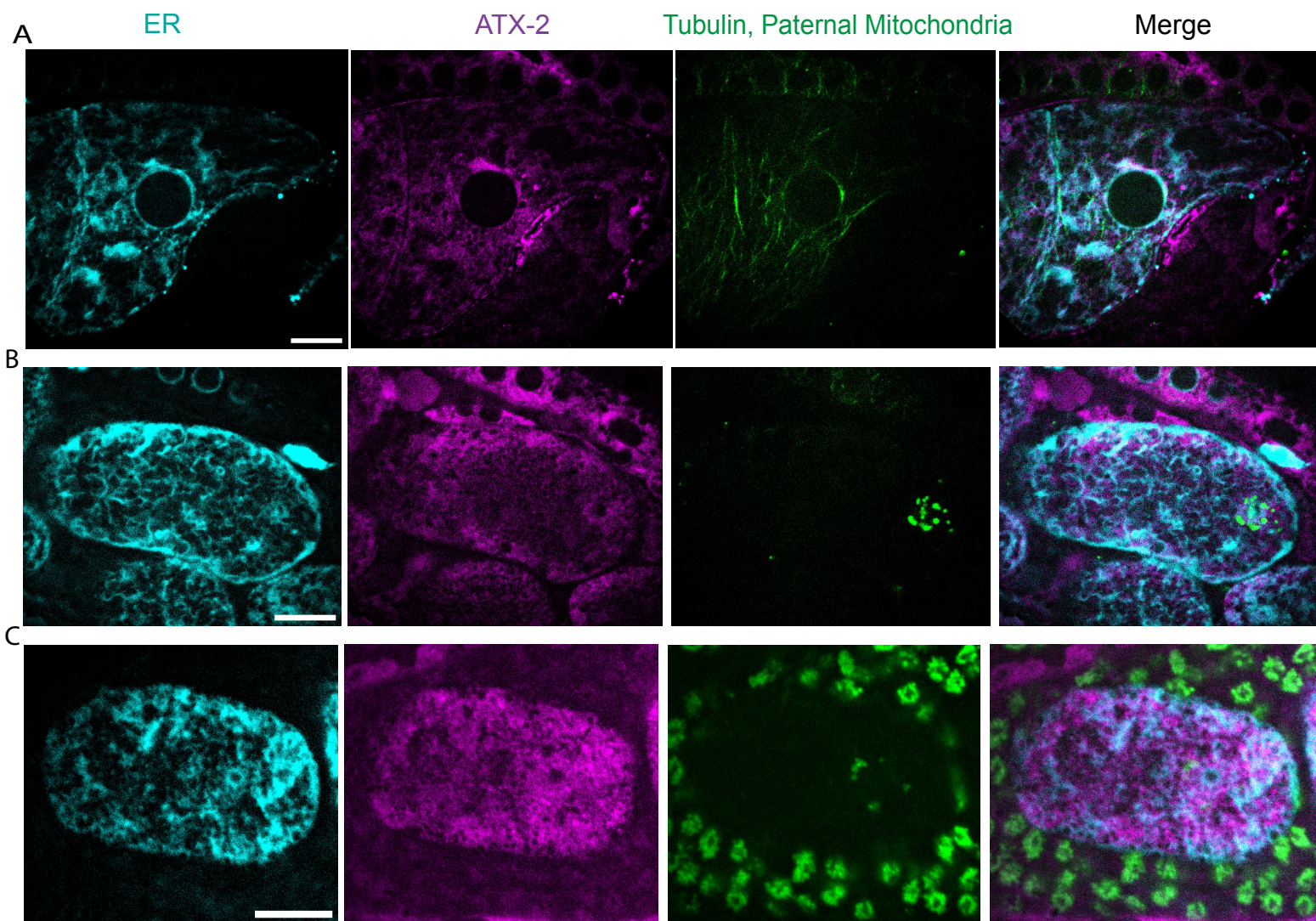

Supplement: Supplement 7 — Figure S2: Localization of ATX-2 in −1 oocyte and +1 meiotic embryo. Deconvolved single plane images from z-stacks acquired on a spinning-disk confocal. A. −1 oocyte (n=10). B. Metaphase meiotic embryo (n=10). C. Anaphase meiotic embryo (n=4). ER labeled with HALO-tag with the signal peptide and ER retention signal from HSP-3. Endogenous ATX-2::AID::GFP. mKate::Tubulin. Paternal mitochondria labeled with SDHC-1::mCherry. All Bars= 10 um. [file media-7.pdf]

A

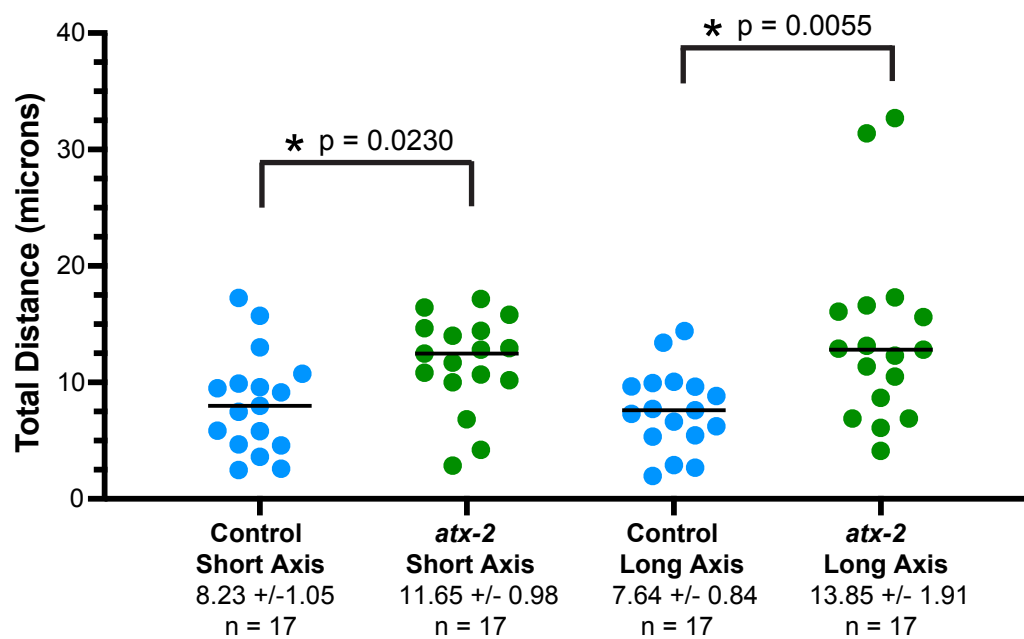

Supplement: Supplement 8 — Figure S3. Cytoplasmic streaming after ATX-2 depletion. Maximum displacement of the sperm contents during any meiotic cell-cycle phase in ATX-2::AID::GFP embryos with or without 1 hr auxin. [file media-8.pdf]
