# Supplementary material for: Katanin, kinesin-13 and ataxin-2 inhibit premature interaction between maternal and paternal genomes in C. elegans zygotes
Source: bioRxiv. 2024 Jun 26:2024.03.12.584242. Originally published 2024 Mar 13. Preprint. [Version 2] doi: 10.1101/2024.03.12.584242 (PMC10979973; doi:10.1101/2024.03.12.584242)
Supplement: Supplement 10 — Table S1 C. elegans strains used in this study. [file media-10.pdf]

Table S1. *C. elegans* strains

Strain Name	Other Name	Genotype Description
N2 Bristol		
FM111	WH327	*unc-119(ed3)* III; *ojls23 [pie-1p::GFP::C34B2.10]*
FM302	CB4108	*fog-2(q71) V*
FM498	BN580	*baf-1(bq12[gfp::baf-1]) III*
FM500		*baf-1(bq12[gfp::baf-1]) III; itIs37 [pie-1p::mCh::H2B::pie-1 3'UTR + unc-119(+)] IV*
FM539	JU2083	*Caenorhabditis macrosperma* wild isolate
FM602	JJ2586	cox-4(zu476[cox-4::eGFP::3xFLAG]) I
FM638		*ojls23 [pie-1p::GFP::C34B2.10]; wjIs76[Cn_unc-119(+); pie-1p::mKate2::tba-2]*
FM647	BCN9071	*vit-2(crg9070[vit-2::gfp]) X*
FM653	KWN724	*sdhc-1(jbm1 [sdhc-1::mCherry]) III; him-5(e1490) V*
FM727		*egxSi126 [mex-5p::hsp-3(aa1-19)::halotag::HDEL::pie-1 3'UTR + unc-119(+)] I; vit-2(crg9070[vit-2::gfp]) X*
FM862		*atx-2(syb5389; ATX-2::AID::GFP) III; ieSi38 [sun-1p::TIR1::mRuby::sun-1 3'UTR + Cbr-unc-119(+)] IV; wjIs76[Cn_unc-119(+); pie-1p::mKate2::tba-2]*
FM932		duSi29{pFM1994[TMCO1::GFP(GLO)::SSPB(nanoGLO)]II} *; [pie-1p-mCh::PH(PLC1delta1) + unc-119(+)]V*
FM956		*ojls23; wjIs76[Cn_unc-119(+); pie-1p::mKate2::tba-2]; atx-2(syb5389; atx-2::AID::GFP); ieSi38 [sun-1p::TIR1::mRuby::sun-1 3'UTR + Cbr-unc-119(+)] IV*
