# Supplementary material for: Katanin, kinesin-13 and ataxin-2 inhibit premature interaction between maternal and paternal genomes in C. elegans zygotes
Source: bioRxiv. 2024 Mar 13:2024.03.12.584242. Preprint. [Version 1] doi: 10.1101/2024.03.12.584242 (PMC10979973; doi:10.1101/2024.03.12.584242)
Supplement: Supplementary file 1 [file NIHPP2024.03.12.584242v1-supplement-6.pdf]

bioRxiv preprint doi: https://doi.org/10.1101/2024.03.12.584242; this version posted March 13, 2024. The copyright holder for this preprint (which was not certified by peer review) is the author/funder, who has granted bioRxiv a license to display the preprint in perpetuity. It is made available under aCC-BY 4.0 International license.

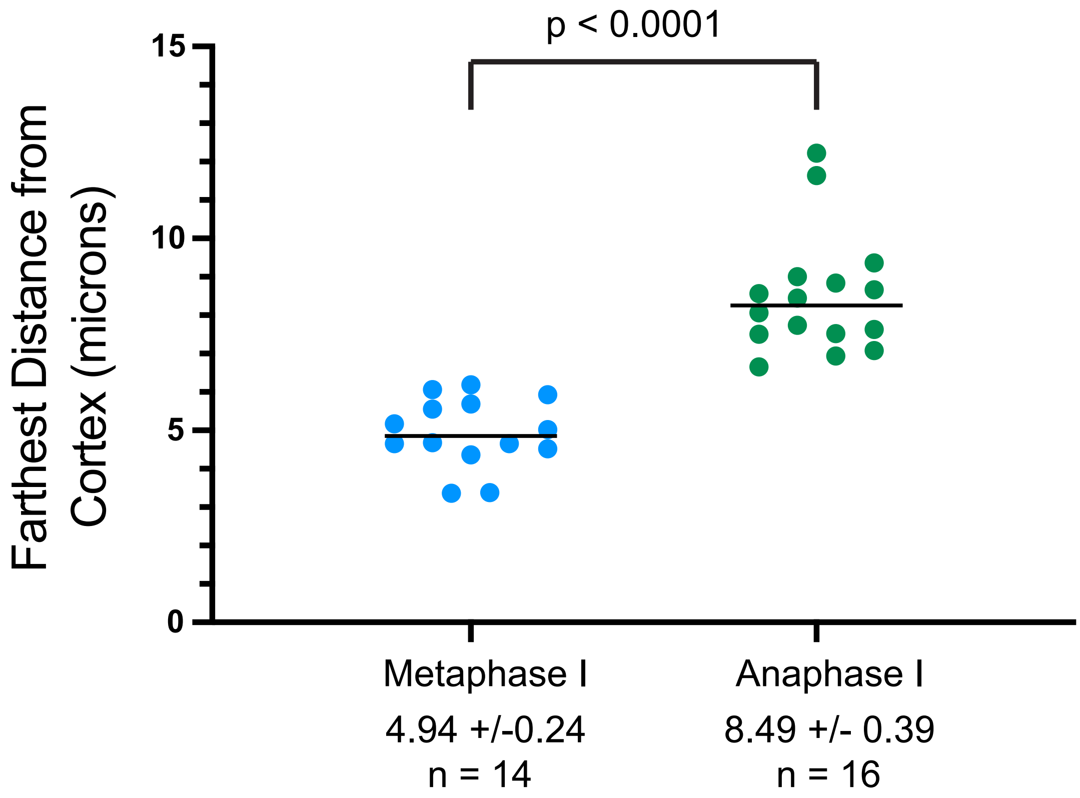

Figure S1. Distance of the sperm contents from the cortex of control embryos at metaphase I vs anaphase I.

bioRxiv preprint doi: https://doi.org/10.1101/2024.03.12.584242; this version posted March 13, 2024. The copyright holder for this preprint
(which was not certified by peer review) is the author/funder, who has granted bioRxiv a license to display the preprint in perpetuity. It is made
available under aCC-BY 4.0 International license.

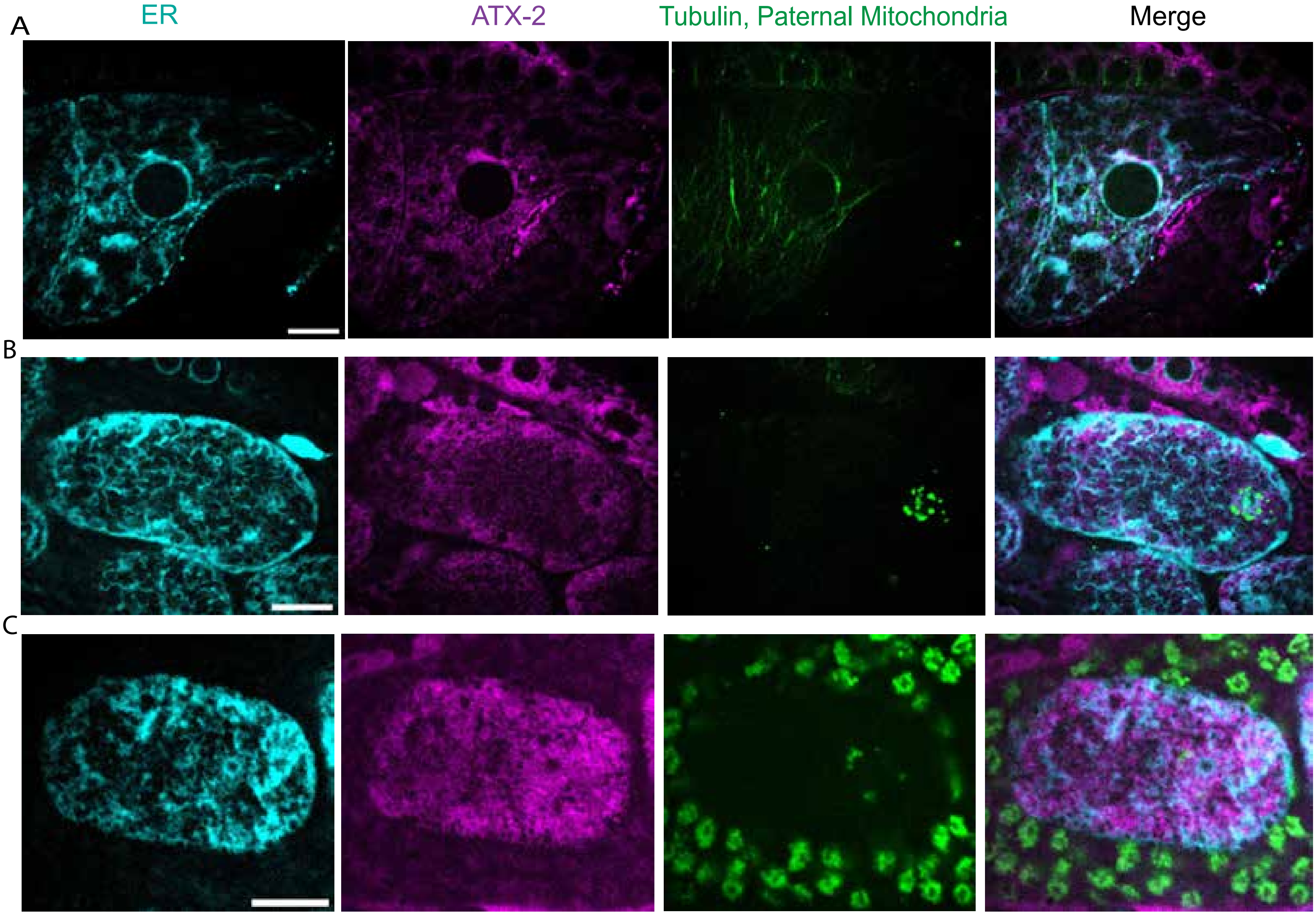

Figure S2: Localization of ATX-2 in -1 oocyte and +1 meiotic embryo. Deconvolved single plane images from z- stacks acquired on a spinning-disk confocal. A. -1 oocyte (n=10). B. Metaphase meiotic embryo (n=10). C. Anaphase meiotic embryo (n=4). ER labeled with HALO-tag with the signal peptide and ER retention signal from HSP-3. Endogenous ATX-2::AID::GFP. mKate::Tubulin. Paternal mitochondria labeled with SDHC-1::mCherry. All Bars= 10um.

bioRxiv preprint doi: https://doi.org/10.1101/2024.03.12.584242; this version posted March 13, 2024. The copyright holder for this preprint (which was not certified by peer review) is the author/funder, who has granted bioRxiv a license to display the preprint in perpetuity. It is made available under aCC-BY 4.0 International license.

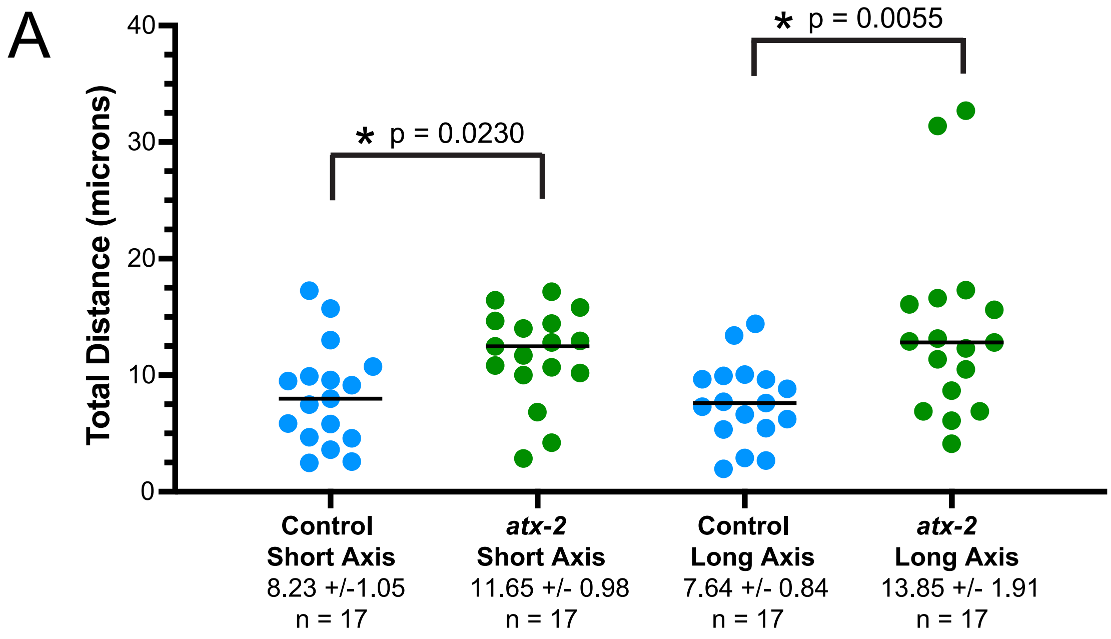

Figure S3. Cytoplasmic streaming after ATX-2 depletion. Maximum displacement of the sperm contents during any meiotic cell-cycle phase in ATX-2::AID::GFP embryos with or without 1 hr auxin.
